# Perspectives on Adrenal Tumor Surgery

**DOI:** 10.3390/medicina62010003

**Published:** 2025-12-19

**Authors:** Catalin Baston, Andreea Parosanu, Oana Moldoveanu, Lucas Discalicău, Pavel Visinescu, Andrei Precup, Ioanel Sinescu

**Affiliations:** 1 Faculty of Medicine, Carol Davila University of Medicine and Pharmacy, 8 Sanitary Heroes Boulevard, 050474 Bucharest, Romania; catalin.baston@umfcd.ro (C.B.); oana.moldoveanu@umfcd.ro (O.M.); ioanel.sinescu@umfcd.ro (I.S.); 2 Fundeni Clinical Institute, 022328 Bucharest, Romania; pavel.visinescu@drd.umfcd.ro (P.V.); andrei.precup@yaho.com (A.P.); 3 Elias University Emergency Hospital, 011461 Bucharest, Romania

**Keywords:** adrenal tumor, adenoma, malign primary, open surgery, laparoscopic adrenalectomy

## Abstract

*Background and Objectives*: Adrenal gland tumors are frequently discovered incidentally. They remain challenging to evaluate because of their heterogeneous nature and overlapping imaging characteristics. Surgical resection continues to represent the primary treatment option for both benign and malignant lesions. This study aimed to characterize the clinical, demographic, and pathological features of adrenal tumors and to assess surgical management patterns in a tertiary referral center. *Materials and Methods:* A retrospective analysis was conducted on 112 patients who underwent adrenalectomy between 2015 and 2022. Demographic, clinical, radiological, and surgical data were reviewed. Histopathological findings were classified as benign tumors, primary adrenal malignancies, or adrenal metastases. Both laparoscopic adrenalectomy and open surgery were performed. The operative approach was determined by tumor characteristics and oncologic considerations. *Results*: Among the 112 patients, 48% had benign adrenal tumors, 32% had adrenal metastases, and 19.6% were diagnosed with primary adrenal malignancies. Most patients with adrenocortical carcinoma were women over 55 years of age. Benign lesions were predominantly managed with simple adrenalectomy and minimally invasive techniques, while malignant tumors frequently required complex oncologic resections and open surgical approaches. Distinct metastatic patterns were observed, with renal cell carcinoma representing the most common primary source of adrenal metastasis. *Conclusions*: Adrenal tumors demonstrate marked demographic and pathological variability. Surgical resection remains essential for definitive diagnosis and treatment, underscoring the importance of tailoring the operative approach. Minimally invasive surgery is appropriate for benign lesions, whereas open adrenalectomy is preferred for malignant or advanced tumors, where surgical expertise is critical to achieving optimal oncologic outcomes.

## 1. Introduction

The prevalence of adrenal tumors, particularly adrenal incidentalomas, has increased substantially in recent years due to the widespread use of high-resolution imaging techniques. Current epidemiological data indicate a prevalence between 4% and 6%, although reported rates vary according to population characteristics and imaging methodology [1,2]. Most adrenal tumors are benign and non-functioning, accounting for approximately 80% of cases, whereas malignant lesions, including adrenocortical carcinoma and adrenal metastases, represent less than 1% and are frequently diagnosed at advanced stages because of their nonspecific clinical features [3].

Adrenal tumors constitute a highly heterogeneous group of lesions, differing in origin, hormonal activity, biological behavior, and prognostic significance. This heterogeneity creates substantial diagnostic challenges, particularly in distinguishing benign from malignant lesions preoperatively. Despite imaging advances, radiological assessment often remains inconclusive. No single imaging characteristic reliably differentiates all malignant tumors from benign ones. Consequently, clinical decision-making may be complex, especially in cases of small, asymptomatic, or indeterminate masses [4].

Surgical management represents a crucial component in the evaluation and treatment of adrenal tumors. Adrenalectomy not only provides definitive therapeutic benefit for most hormonally active or malignant tumors but also remains the only method capable of offering complete histopathological assessment, which is essential for establishing a definitive diagnosis. Minimally invasive techniques, such as laparoscopic or robotic adrenalectomy, are now widely adopted for benign lesions, whereas open approaches remain recommended for large, invasive, or highly suspicious masses. Nevertheless, debates persist regarding the indication for surgery in small non-functioning incidentalomas, the optimal surveillance strategy, and the preferred surgical technique [5,6].

Given the heterogeneity of adrenal tumors and the uncertainty surrounding optimal management, a detailed understanding of current clinical patterns is essential. The purpose of this study is primarily descriptive, aiming to characterize the demographic, pathological, and surgical patterns observed in a tertiary oncologic referral center. Such institutions inherently receive a higher proportion of complex and malignant adrenal tumors, and understanding these patterns is essential for identifying gaps in current diagnostic pathways and for guiding future prospective research rather than proposing changes to clinical practice.

## 2. Materials and Methods

This retrospective observational study included patients who underwent adrenalectomy between January 2015 and December 2022 at a tertiary urologic referral center. A total of 131 consecutive cases were initially identified. The study was approved by the Institutional Review Board of the National Medical Center, and all procedures were conducted in accordance with institutional ethical standards. Twelve cases of hormonally active adrenal tumors were excluded. These patients follow a separate multidisciplinary endocrine pathway in our institution, which includes specific preoperative management (such as hormonal control and alpha-blockade). Their timing of surgery and guideline-driven indications differ from the oncologic decision-making applied to non-functional adrenal masses. Therefore, 119 patients were initially retained for screening. Of these, 7 were excluded due to incomplete clinical or histopathological data. Consequently, the final study cohort consisted of 112 patients.

### 2.1. Patient Selection and Preoperative Evaluation

Patients were stratified according to sex, age (<55 years or ≥55 years), and tumor laterality (right or left). All individuals underwent preoperative clinical, biochemical, and radiological evaluation. Imaging assessment was performed by radiologists specialized in oncologic urologic imaging and included contrast-enhanced computed tomography (CT) in all cases, with magnetic resonance imaging (MRI) or positron emission tomography (PET-CT) performed selectively in cases of indeterminate or suspicious lesions. In this study, however, detailed imaging parameters (e.g., HU values, washout characteristics, MRI signal, PET-CT uptake) could not be systematically analyzed because they were not consistently available in a standardized format across all patients in this retrospective cohort. For this reason, imaging data were used descriptively to guide surgical decision-making but were not included in the statistical analysis.

For benign, non-functioning adrenal tumors, surgical indication was established based on a combination of radiological, clinical, and oncologic criteria. Adrenalectomy was recommended for lesions exceeding 4–6 cm in diameter, for masses demonstrating interval growth on serial imaging, or for tumors with atypical radiologic characteristics (density > 10 HU, heterogeneous composition, irregular margins, or insufficient washout suggestive of non-adenomatous pathology). Surgery was also considered in cases where preoperative assessment could not reliably exclude malignancy or when patients experienced mass-related symptoms. All cases were reviewed in a multidisciplinary setting prior to decision-making.

### 2.2. Surgical Approaches

Two surgical approaches were used: open adrenalectomy and laparoscopic adrenalectomy. Procedures were categorized as simple adrenalectomy, defined as removal of the adrenal gland without involvement of adjacent structures, or as complex oncologic resection, referring to adrenalectomy performed as part of an extended procedure requiring en bloc removal of neighboring organs or tissues. The latter approach was typically indicated in cases of locally invasive or metastatic disease.

The choice of surgical technique was based on tumor size, radiologic characteristics, patient surgical history, and surgeon expertise. Open adrenalectomy was preferred for tumors > 6 cm, lesions with imaging evidence of local invasion, synchronous intra-abdominal malignancies, extensive previous abdominal surgery with anticipated adhesions, or when complex oncologic resection was required. All adrenalectomies for metastatic disease were performed as intentional metastasectomies following multidisciplinary evaluation, given radiologic evidence of solitary or oligometastatic adrenal involvement.

In patients without a documented oncological history, the same risk-adapted criteria were applied. Open surgery was selected for large tumors, for lesions with radiological features suspicious for malignancy or local invasion, or when complex or multivisceral resections were anticipated. An open approach was also preferred in patients with extensive prior abdominal surgery and expected adhesions. Conversely, smaller, radiologically benign lesions in patients without oncologic history were managed primarily by laparoscopic adrenalectomy.

### 2.3. Histopathological Assessment

**Figure 1:**
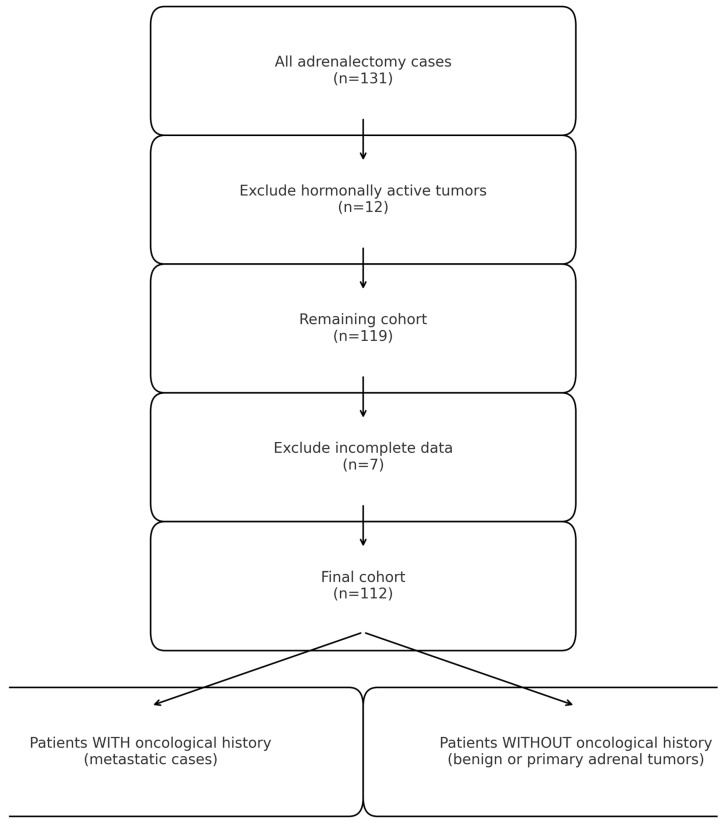
Overview of patient stratification.

All resected specimens underwent formal histopathological evaluation within the Department of Urology’s pathology service. Tumors were classified into three major categories: primary benign adrenal tumors (BP), primary malignant adrenal tumors (MP), and secondary malignant adrenal tumors (MS) (metastatic lesions from extra-adrenal primaries). Among the 119 included patients, 54 (48.2%) had benign tumors (including 50 adenomas, 3 ganglioneuromas, and 1 schwannoma). Secondary malignant tumors represented 32.1% of cases, while 19.6% were primary adrenal malignancies (Figure 1).

### 2.4. Statistical Analysis 

Continuous variables were compared using ANOVA or *t*-tests, while categorical variables were analyzed using chi-square or Fisher’s exact tests as appropriate. All data analyses were performed using SPSS version 23.0.

## 3. Results

### 3.1. Demographic and Clinical Characteristics of the Study Population

A total of 112 patients underwent adrenalectomy for adrenal masses during the study period. The median age was 62 years (range: 19–76 years). The cohort included an equal distribution of women and men (56 vs. 56). Histopathological evaluation classified 54 cases (48.2%) as PB, 36 (32.1%) as MS and 22 (19.6%) as primary malignant tumors (MP). Detailed demographic and clinical characteristics are presented in Table 1.

Age showed a significant association with histopathological outcome (*p* = 0.018). Malignant tumors (MS and MP) were predominantly diagnosed in patients older than 55 years, while benign tumors were more common in younger individuals.

Sex distribution differed significantly between groups (*p* = 0.004). Benign primary and metastatic secondary tumors were more prevalent in men, whereas primary malignant tumors were more frequently observed in women.

Tumor laterality showed a similar distribution between the right and left adrenal glands across all histological subgroups and did not demonstrate a significant association with benign or malignant outcomes (*p* = 0.535). Laterality was included only as a descriptive variable, and in our cohort it did not contribute to distinguishing between histological categories or to the predictive modeling of malignancy.

### 3.2. Surgical Management and Approach

Marked differences in surgical management were observed across histopathological categories (*p* = 0.001). Standard adrenalectomy was the most common intervention overall and was especially frequent in PB tumors (40 of 54 cases). In contrast, MS and MP tumors more often required adrenalectomy combined with nephrectomy or complex oncologic resections. These procedures reflected local invasion or the presence of synchronous malignancies.

The surgical approach also varied significantly depending on tumor type (*p* = 0.001). Laparoscopic adrenalectomy was primarily employed for PB lesions, consistent with their smaller size and benign imaging characteristics. In malignant tumors, however, open surgery was overwhelmingly preferred, being performed in 34 of 36 MS cases and in 19 of 22 MP cases. This reflects the need for wider exposure, better control of vascular structures, and the possibility of achieving en bloc resection in patients with locally advanced or metastatic disease.

### 3.3. Comparative Analysis and Predictors of Malignancy

A comparative analysis between benign (PB) and malignant adrenal tumors (combined MP/MS) revealed several significant differences (Table 2). Patients with malignant tumors were significantly older than those with benign lesions, with a mean age of 63 ± 8 years compared to 55 ± 10 years (*p* = 0.001). Surgical approach also differed between groups: Open adrenalectomy was significantly more common in malignant tumors (89%) compared with benign lesions (61%, *p* = 0.022). Complex oncologic procedures were also more common in malignant cases. They occurred in 25% of MP/MS tumors compared with only 10% of PB tumors (*p* = 0.008). A tendency toward increased bilateral tumor involvement was observed in malignant cases, although this difference did not reach statistical significance (*p* = 0.082).

Multivariate logistic regression further confirmed age, surgical type, and surgical management as independent predictors of malignancy. Each additional year of age increased the odds of malignancy by 7% (*OR* 1.07; 95% *CI* 1.03–1.12; *p* = 0.001). Similarly, undergoing open surgery was strongly associated with malignant histology (*OR* 2.02; 95% *CI* 1.06–3.42; *p* = 0.031), while complex adrenalectomy significantly increased the likelihood of malignancy (*OR* 2.23; 95% *CI* 1.05–4.75; *p* = 0.036). Tumor location, however, did not emerge as an independent predictor in the adjusted model (*p* = 0.064).

### 3.4. Clinical Characteristics by Histological Subtype

**Figure 2:**
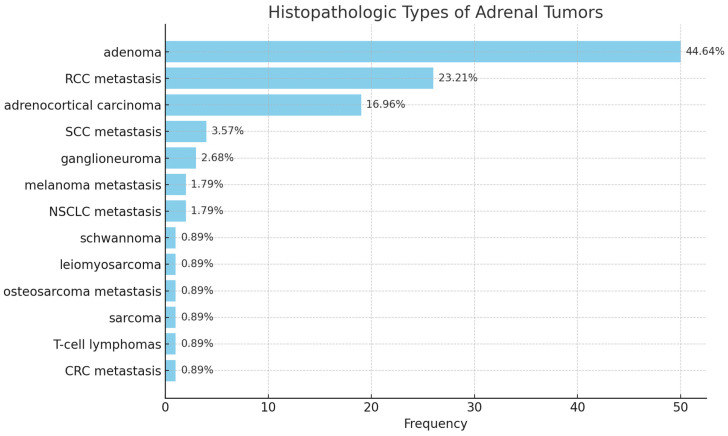
Histological subtypes.

Histopathological analysis revealed that 48.2% of patients (*n* = 54) presented with benign adrenal tumors, consisting of 50 adenomas, 3 ganglioneuromas, and 1 schwannoma. The remaining cases included 32.1% of patients with previous or synchronous extra-adrenal malignancies (secondary malignant tumors) and 19.6% with primary adrenal malignancies (Figure 2). Among patients diagnosed with adrenal metastases, renal cell carcinoma (RCC) represented the most frequent primary origin, followed by squamous cell carcinoma (SCC), melanoma, colorectal cancer (CRC), and bone malignancies. This distribution reflects typical metastatic pathways to the adrenal gland, with RCC being particularly prone to adrenal involvement due to its aggressive hematogenous dissemination.

In all patients with adrenal metastases, the adrenalectomy represented a deliberate metastasectomy rather than an incidental finding. Each case had a previously established oncologic diagnosis, and adrenal involvement was either radiologically detected before surgery or identified intraoperatively in the context of synchronous renal tumors. No adrenal metastasis was discovered unexpectedly at histopathological examination in patients without prior oncologic history.

Among patients with adrenal metastases, renal cell carcinoma (RCC) represented the most frequent primary origin, accounting for 26 cases (23.2%). Patients with RCC metastases had a mean age of 62.7 years (range: 50–75 years), indicating that adrenal involvement occurs predominantly in middle-aged to elderly individuals (Table 3). Metastatic deposits in RCC were almost equally distributed between the right (*n* = 12) and left adrenal gland (*n* = 13), with one case presenting bilateral disease.

In contrast, metastases from other primary tumors, including colorectal cancer (CRC), melanoma, squamous cell carcinoma (SCC), non-small cell lung cancer (NSCLC), and osteosarcoma, were exclusively unilateral and most commonly affected the right adrenal gland. A male predominance was observed across nearly all metastatic categories. This finding may suggest a sex-related predisposition to adrenal metastases.

Regarding surgical management, open adrenalectomy was the approach most frequently performed in patients with adrenal metastases, reflecting the need for optimal access and oncological control in cases of suspected or confirmed metastatic involvement. Laparoscopic adrenalectomy was reserved for only a minority of patients, typically those with smaller or less complex lesions.

Within primary adrenal malignancies, adrenocortical carcinoma (ACC) was by far the most prevalent diagnosis, accounting for 16.96% of all cases (*n* = 19), while other malignant entities—including leiomyosarcoma, T-cell lymphoma, and sarcoma—were exceptionally rare, each representing less than 1% of the cohort (Table 4). The mean age of patients with ACC was 59 years, reflecting the typical presentation of this highly aggressive neoplasm in late middle age.

A marked female predominance was observed in ACC, with women representing approximately two-thirds of affected patients (14 females vs. 5 males). Regarding tumor laterality, ACC cases were evenly distributed between the right and left adrenal glands, and a single patient presented bilateral involvement.

Open adrenalectomy was the primary surgical approach for nearly all primary adrenal malignancies, reflecting the need for comprehensive oncologic resection and optimal visualization in cases with large or invasive tumors. However, two patients with ACC underwent laparoscopic adrenalectomy, demonstrating that minimally invasive techniques may be selectively feasible in well-chosen cases with limited tumor size and no radiological evidence of local invasion.

## 4. Discussion

Adrenal tumors represent a highly heterogeneous group of lesions, encompassing benign, functional, malignant, and metastatic entities, each with distinct clinical behavior and therapeutic implications. The increasing availability of cross-sectional imaging has raised the detection rate of adrenal masses. Recent studies estimate a prevalence of about 10% among patients undergoing abdominal imaging, especially in those older than 55 years [7,8]. Our findings align with this pattern, as most patients in our cohort were diagnosed in their sixth decade of life.

The differential diagnosis of adrenal masses is broad and includes conditions such as adrenocortical hyperplasia, adenomas, myelolipomas, pheochromocytomas, adrenocortical carcinoma, metastases, hemorrhage, and granulomatous diseases such as tuberculosis [9,10,11]. Among benign lesions, adrenocortical adenomas account for up to 85% of cases. They generally follow an indolent course and require intervention only when hormonally active or when they show significant growth [12,13]. In contrast, ACC is a rare but highly aggressive malignancy, with an incidence of 0.5–2 cases per million per year and a poor prognosis in advanced stages [14,15]. Preoperative imaging assessment was not prospectively stratified by degrees of suspicion, which limits the ability to quantify discrepancies between radiologic impressions and final histopathological findings. Nonetheless, the substantial overlap in imaging characteristics reinforces the pivotal role of surgical excision in establishing a definitive diagnosis.

Interestingly, our study found that nearly half of the surgically treated adrenal tumors were malignant. This rate is considerably higher than that reported in population-level studies and likely reflects referral bias, as our high-volume tertiary oncologic center predominantly receives patients with complex, suspicious, or advanced disease. Nonetheless, the demographic trends observed in our cohort, such as the predominance of women among ACC patients and the older age of those with malignant tumors, echo findings from previous epidemiological studies [16,17,18,19].

In our cohort, benign non-functioning adrenal tumors were selected for surgery primarily when size, interval growth, or atypical radiological features raised concern for malignancy. These criteria align with current international recommendations and reflect the practical necessity of surgical excision when imaging alone cannot provide a definitive distinction between benign and malignant lesions. This approach explains why a relevant proportion of non-functioning benign tumors in our series underwent operative management.

Importantly, all adrenal metastases in this cohort were managed as intentional metastasectomies. Each surgical indication was supported by multidisciplinary consensus, with adrenalectomy performed for patients with isolated or oligometastatic disease or in the setting of complex synchronous oncologic surgery, particularly RCC. No incidental metastases were identified in patients without a known primary malignancy, reinforcing the oncologic nature of our surgically enriched population.

### 4.1. Adrenal Metastases: Patterns and Clinical Implications

Adrenal metastases represent a distinct and clinically relevant subset of adrenal tumors. Chen et al. reported metastatic involvement in about 30% of adrenal lesions in surgical series [20]. Similarly, our study found adrenal metastases in 32.1% of patients. Lung cancer, breast cancer, and RCC are commonly reported sources of adrenal metastases in the general oncology population [21,22,23]. In our cohort, however, RCC was the predominant primary tumor. This finding may reflect institutional specialization, given the oncologic urology focus of our center.

Consistent with established epidemiological evidence, advanced age was strongly associated with malignant or metastatic adrenal disease, and age remained an independent predictor of malignancy after adjustment for clinical and surgical variables [12,13]. This pattern was especially evident among patients with RCC metastases, who had a median age of 62 years and a predominantly male profile, aligning with the known epidemiology of RCC.

Metastatic spread in RCC was nearly evenly distributed between the right and left adrenal glands, with three cases of bilateral involvement, whereas metastases from CRC, melanoma, NSCLC, and osteosarcoma were exclusively unilateral. Although laterality has been discussed in previous studies, particularly in RCC, due to potential anatomical differences in venous drainage [16,17], tumor side did not reach statistical significance in our cohort and did not contribute meaningfully to malignancy prediction (*p* = 0.082). Therefore, laterality was included solely as a descriptive variable, in line with reporting standards in adrenal surgery series.

### 4.2. Diagnostic Challenges and Predictors of Malignancy

Differentiating benign from malignant adrenal masses remains challenging despite advances in imaging. Size, radiologic phenotype, and washout characteristics provide useful clues, but significant overlap persists. This is especially true for atypical adenomas, metastases, and small ACCs. Our multivariate analysis identified age, surgical type, and surgical complexity as independent predictors of malignancy, with age being the strongest predictor. Each additional year increased the probability of a malignant diagnosis by 7%, underscoring the importance of age-based risk stratification.

### 4.3. Surgical Management: Laparoscopic vs. Open Adrenalectomy

Surgical excision remains the cornerstone of management for most adrenal tumors, serving both diagnostic and therapeutic purposes. In our series, laparoscopic adrenalectomy was the preferred approach in over 75% of cases, particularly for benign lesions, a finding in line with international guidelines recommending minimally invasive surgery as the gold standard for small, unilateral, and radiologically benign tumors [24,25,26,27,28,29].

Open adrenalectomy remains the recommended approach for large tumors or for cases with suspected local invasion. It is also preferred in patients with extensive prior abdominal surgery and in malignant tumors that require complex oncologic resections [30,31,32]. Our results fully reflect these criteria: open surgery was selected for tumors larger than 6 cm, lesions with radiologic suspicion of invasion, synchronous malignancies, and when multivisceral resection or combined adrenalectomy–nephrectomy was needed. Notably, 25% of our patients required extended oncologic procedures, and another 25% underwent combined adrenalectomy and nephrectomy, highlighting the high complexity profile of cases treated in tertiary oncologic centers.

The use of open adrenalectomy in patients without a prior oncologic history reflects these same selection criteria rather than an intrinsic prognostic value of the surgical approach itself. In this subgroup, open surgery was reserved for large or radiologically suspicious tumors and for technically demanding cases, while clearly benign-appearing lesions were treated laparoscopically. Consequently, the observed association between open adrenalectomy and malignant histology is largely driven by preoperative selection and should be interpreted with caution.

Laparoscopy is generally contraindicated in ACC and invasive tumors. However, two patients in our study underwent successful laparoscopic resection for presumed early-stage ACC. This suggests that minimally invasive surgery may be feasible in carefully selected cases with limited tumor size and no radiological evidence of invasion. However, these exceptions should be approached with caution and ideally reserved for centers with significant adrenal and oncologic surgical experience.

We acknowledge that the association observed between open or complex adrenalectomy and malignant histology may be influenced by preoperative imaging and clinical suspicion. In our tertiary setting, open or extended procedures were appropriately selected for lesions with features suggestive of higher oncologic risk. Thus, the surgical approach reflects careful preoperative judgment rather than acting as an independent determinant of malignancy. The persistence of the association after adjustment likely reflects the inherent limitations of current diagnostic tools, and we appreciate the reviewer’s comment for helping us clarify this point more explicitly.

### 4.4. Limitations and Future Directions

The present study provides a comprehensive overview of surgically managed adrenal tumors in a tertiary oncologic setting, but several limitations must be acknowledged. The retrospective design introduces inherent selection bias and limits causal inference. Because our center serves as a referral institution, malignant and complex cases were overrepresented compared with the general incidentaloma population. This referral bias likely accounts for the higher proportion of metastatic and primary adrenal malignancies identified.

The disproportionately high proportion of malignant tumors (both primary and metastatic) compared with population-based incidentaloma studies represents one of the distinctive features of our cohort. This finding reflects institutional referral patterns but also underscores the complexity of cases treated in tertiary oncologic centers. Moreover, the balanced right–left distribution of RCC adrenal metastases and the higher-than-usual female predominance in ACC constitute additional deviations from classical epidemiological trends, warranting further investigation in larger multicenter datasets.

Hormonally active adrenal tumors were excluded to preserve cohort homogeneity, as these patients follow distinct endocrine-driven pathways with different preoperative and surgical considerations. Additionally, data on hormonal status, postoperative complications, long-term survival, and recurrence were inconsistently available, restricting our ability to evaluate long-term oncologic outcomes.

Another limitation concerns the assessment of surgical approach. Laparoscopic adrenalectomy was used almost exclusively for benign lesions, whereas malignant tumors generally required open surgery due to size, invasion, or the need for extended resections. The small number of minimally invasive procedures in malignant cases precluded meaningful comparison, reflecting strong selection bias and heterogeneous follow-up.

Importantly, the retrospective nature of the study and the absence of standardized radiologic parameters, including attenuation values, washout indices, and uniform MRI reporting, limited our ability to incorporate imaging predictors into malignancy modeling. Likewise, incomplete documentation of postoperative outcomes restricted meaningful evaluation of surgical efficacy. Together, these constraints significantly reduce the predictive power and broader clinical impact of the present work, underscoring the need for harmonized imaging protocols, systematic surgical outcome reporting, and prospective multicenter databases to support more robust risk stratification and evidence-based decision-making.

Further research should also clarify the role of minimally invasive adrenalectomy in carefully selected malignant cases, including early-stage adrenocortical carcinoma and solitary adrenal metastases.

## 5. Conclusions

This study provides insight into the characteristics of a surgically enriched adrenal tumor population managed in a tertiary oncologic center, a setting that differs substantially from general incidentaloma cohorts. Within this context, age, surgical complexity, and the need for open adrenalectomy emerged as independent predictors of malignancy, reflecting real-world patterns encountered in high-risk adrenal tumors.

Adrenal lesions demonstrated substantial demographic and pathological variability across benign, primary malignant, and metastatic subtypes. Malignant tumors were more frequent in older patients and more often required open or extended oncologic procedures, consistent with their larger size and invasive behaviour. In contrast, benign lesions were predominantly treated with minimally invasive adrenalectomy.

These findings emphasize the need for individualized evaluation and tailored operative strategies based on clinical, radiologic, and pathological characteristics. Prospective, multicenter studies are warranted to refine preoperative risk stratification and to establish evidence-based criteria for selecting the optimal surgical approach.

Therefore, these findings should be interpreted as descriptive real-world evidence derived from a high-complexity oncologic context, serving primarily as a foundation for future investigative efforts rather than as a basis for immediate modifications in clinical practice.

## Figures and Tables

**Table 1:** Patient demographics and clinical features.

Parameter	Total (*n* = 112)	Primary Benign (*n* = 54)	Metastatic (*n* = 36)	Primary Malignant (*n* = 22)	*p*-Value
Age, years (mean ± SD)	62 ± 10	55 ± 10	63 ± 8	64 ± 9	0.018
Age ≥ 55 years, *n* (%)	86 (77%)	18 (33%)	33 (92%)	18 (82%)	
Sex, *n* (%)					0.004
Female	56 (50%)	20 (37%)	10 (28%)	26 (59%)	
Male	56 (50%)	34 (63%)	26 (72%)	12 (41%)	
Tumor laterality, *n* (%)					0.535
Right	51 (46%)	26 (48%)	17 (47%)	8 (36%)	
Left	52 (46%)	25 (46%)	16 (44%)	11 (50%)	
Bilateral	8 (7%)	3 (6%)	3 (8%)	2 (9%)	
Surgical type, *n* (%)					0.001
Standard adrenalectomy	69 (62%)	40 (74%)	11 (31%)	8 (36%)	
Adrenalectomy + nephrectomy	25 (22%)	6 (11%)	10 (28%)	9 (41%)	
Complex oncologic resection	17 (15%)	8 (15%)	9 (25%)	5 (23%)	
Surgical approach, *n* (%)					0.001
Open	86 (77%)	33 (61%)	34 (94%)	19 (86%)	
Laparoscopic	26 (23%)	21 (39%)	2 (6%)	3 (14%)	

**Table 2:** Risk factors for histological differentiation.

Parameter	Benign (PB) *n* = 60	Malignant (MP/MS) *n* = 40	Univariate *p*-Value	Multivariate *OR* (95% *CI*)	Multivariate *p*-Value
Age (years)					
Mean ± SD	55 ± 10	63 ± 8	*p* = 0.001	1.07 (1.03–1.12)	*p* = 0.001
Surgical approach, *n* (%)					
Open surgery	85%	70%	*p* = 0.022	2.02 (1.06–3.42)	*p* = 0.031
Laparoscopic surgery	15%	30%		Reference	
Tumor laterality, *n* (%)					
Right	65%	55%	*p* = 0.082	1.64 (0.97–2.79)	*p* = 0.064
Left	35%	40%			
Bilateral	0%	5%			
Surgical management, *n* (%)					
Standard adrenalectomy	90%	75%	*p* = 0.008	2.23 (1.05–4.75)	*p* = 0.036
Complex oncologic procedure	10%	25%			

**Table 3:** Patient characteristics with adrenal metastases.

Primary Cancer	*n* (%)	Mean Age (Range)	Gender (M/F)	Surgical Approach (Open/Lap)	Laterality (Right/Left/Bilateral)
Renal cell carcinoma (RCC)	26 (72%)	62.7 (50–75)	17/9	24/2	12/13/1
Colorectal cancer (CRC)	1 (3%)	73	1/0	1/0	1/0/0
Melanoma	2 (6%)	65	2/0	2/0	2/0/0
Squamous cell carcinoma (SCC)	4 (11%)	60.8	3/1	3/1	0/3/0
Non-small cell lung cancer (NSCLC)	1 (3%)	71	1/0	1/0	0/1/0
Osteosarcoma	1 (3%)	45	1/0	1/0	1/0/0

**Table 4:** Clinical characteristics by primary adrenal malignancy.

Diagnosis	*n* (%)	Mean Age	Gender (M/F)	Surgical Approach (Open/Lap)	Laterality (Right/Left/Bilateral)
Adrenocortical carcinoma (ACC)	19 (86%)	59	5/14	17/2	9/9/1
Leiomyosarcoma	1 (4%)	69	1/0	1/0	0/1/0
T-cell lymphoma	1 (4%)	61	0/1	1/0	0/0/1
Sarcoma (unspecified)	1 (4%)	58	0/1	1/0	1/0/0

## Data Availability

The original contributions presented in this study are included in the article. Further inquiries can be directed to the corresponding author.

## Abbreviations

The following abbreviations are used in this manuscript:

MP	Malignant Primary
MS	Malignant Secondary
BP	Primary Benign
RCC	Renal Cell Carcinoma
CRC	Colorectal Cancer
SCC	Squamous Cell Carcinoma
NSCLC	Non-Small Cell Lung Cancer
SCLC	Small Cell Lung Carcinoma
ACC	Adrenocortical Carcinoma
